# Hybrid identification in *Nothofagus* subgenus using high resolution melting with ITS and trnL approach

**DOI:** 10.7717/peerj.6779

**Published:** 2019-05-09

**Authors:** Jaime Solano, Leonardo Anabalón, Francisco Encina, Carlos Esse, Diego Penneckamp

**Affiliations:** 1 Departamento de Ciencias Agropecuarias y Acuícolas, Facultad de Recursos Naturales, Universidad Católica de Temuco, Chile, Universidad Católica de Temuco, Temuco, Chile; 2 Departamento de Ciencias Biológicas y Químicas, Facultad de Recursos Naturales, Universidad Católica de Temuco, Chile, Universidad Católica de Temuco, Temuco, Chile; 3 Departamento de Ciencias Ambientales, Facultad de Recursos Naturales, Universidad Católica de Temuco, Chile, Universidad Católica de Temuco, Temuco, Chile; 4 Instituto de Estudios del Hábitat (IEH), Universidad Autónoma de Chile, Centro de Investigación Multidisciplinario de la Araucanía (CIMA), Universidad Autónoma de Chile, Universidad Autónoma de Chile, Temuco, Chile; 5 Facultad de Ciencias Forestales y Recursos Naturales, Universidad Austral de Chile, Universidad Austral de Chile, Valdivia, Chile

**Keywords:** HRM, ITS region, trnL locus, *Nothofagus*, *Nothofagus* subgenus

## Abstract

The genus *Nothofagus* is the main component of southern South American temperate forests. The 40 *Nothofagus* species, evergreen and deciduous, and some natural hybrids are spread among Central and Southern Chile, Argentina, New Zealand, Australia, New Guinea and New Caledonia. *Nothofagus nervosa*, *Nothofagus obliqua* and *Nothofagus dombeyi* are potentially very important timber producers due to their high wood quality and relative fast growth; however, indiscriminate logging has degraded vast areas the Chilean forest causing a serious state of deterioration of their genetic resource. The South of Chile has a large area covered by secondary forests of *Nothofagus dombeyi*. These forests have a high diversity of species, large amount of biomass and high silvicultural potential. This work shows a case of hybrid identification in *Nothofagus* subgenus in different secondary forests of Chile, using high resolution melting. Unknown samples of* Nothofagus* subgenus are genetically distinguishable with the ITS region of *Nothofagus antarctica*, *Nothofagus nitida* and *N. obliqua* species. It was not possible to distinguish between unknown samples of Andean versus coastal origin. Melting curves with ITS approach of unknown material are genetically similar, positioned between *N. dombeyi* and *N. antarctica* and distant from *N. nitida*. The unknown samples are genetically very close to *Nothofagus dombeyi*. This suggests the presence of hybrid individuality between species (*N. dombeyi* × *N. antarctica*) with the possibility of introgression towards the gene pool of *N. antarctica*, producing the deciduous foliage that is both present. The trnL locus has no distinction between the *N. dombeyi* and *N. antarctica* species, since a similar melting curve is present and equal Tm (80.00 °C). The trnL locus cannot be genetically distinguished from one unknown sample of *Nothofagus* to another, as highlighted in this study.

## Introduction

*Nothofagus* (*Nothofagaceae*) species are the main component of southern South American temperate forests. Overexploitation in the past has led to the loss of 40% of the original distribution range (Marchelli et al., 2008). Genetic diversity as well as biological processes shaping the distribution of the genetic variation (e.g., gene flow) constitutes basic knowledge for the implementation of conservation measures, ecological restoration and definition of Evolutionary Significant Units (Marchelli et al., 2008). *Nothofagus* are segregated in four sub-genera, mainly according to cupule morphology and leaf anatomy (Hill & Read, 1991). *Nothofagus* subgenus are *Brassospora* (New Caledonia and New Guinea), *Fuscospora* (New Zealand and South America), *Lophozonia* (Australia, NZ and SA) and *Nothofagus* (southern South America). The clade of this classification has been strongly supported by DNA studies (Martin & Dowd, 1993; Manos, 1997; Heenan & Smissen, 2013) and polen morphology (Fernández et al., 2016). Subgenus *Nothofagus* consists of five woody species, three evergreen (*Nothofagus betuloides* (Mirb.) Oerst., *Nothofagus dombeyi* (Mirb.) Oerst., *Nothofagus nitida* (Phil.) Krasser) and two deciduous (*Nothofagus antarctica* (G. Forst.) Oerst., *Nothofagus pumilio* (Poepp.&Endl)) that are present in diverse forest associations of temperate forest of Argentina and Chile (Manos, 1997), all of them dominant species in their original forest ecosystems (Lusk & Ortega, 2003; Esse et al., 2014). A wide range of molecular biological methods has been developed over the last decade for the identification of organisms, from the generic to individual level. One such region is the ITS (Internal Transcribed Spacer) region. Nuclear ITS sequencing subgenus *Nothofagus* has been used by different authors. Reports showed that *N. pumilio* diverged earlier and that *N. antarctica* is sister to the monophyletic group containing the three evergreens species (*N. betuloides*; *N. dombeyi* and *N. nitida* (Acosta & Premoli, 2010)). Manos (1997), reports systematic *Nothofagus* based rDNA spacer sequences (ITS) and compares the taxonomic congruence with morphology and plastid sequences. Nuclear ribosomal DNA sequences encoding the 5.8s rRNA and two flanking internal transcribed spacers (ITS) provided 95 phylogenetically informative nucleotide sites from a single alignment of ∼588 bases per species (Manos, 1997).

Four monophyletic groups were obtained by Parsimony analysis. In this way the variation produced two equally parsimonious trees. These groups correspond to different pollen type of *Nothofagus* species. Reanalyzes tree of previously reported rbcL sequences and morphological data set with these topologies were compared. There was a high congruence for these three data sets, with topological differences restricted to a location of a few terminal taxa according to the parsimony analysis. Six equally parsimonious trees were produced by morphological and molecular analysis. The agreement of these trees suggest two basal clades within *Nothofagus* that corresponding to tropical *Nothofagus* (subgenus *Brassospora*) of New Guinea and New Caledonia are strongly supported as sister to cool-temperate species of South America (subgenus *Nothofagus*) (Manos, 1997).

The hybridization between *Nothofagus* species in natural habitat and in culture was reported by Donoso, Morales & Romero (1990), Premoli (1996a), Donoso (2004) and Azpilicueta & Gallo (2009). In southern-central Chile *Nothofagus x leonii,* a hybrid between *Nothofagus obliqua* (Mirb.) Oerst. and *Nothofagus glauca* (Phil.) Krasser. Ann. K.K. Naturhist. Hofmus (subgenus *Lophozonia*) developed in the habitat overlapped by the parental, and has ecological importance forming stands in the forest (Donoso & Landrum, 1979; Donoso, 2006). Further natural hybrid trees between *N. obliqua* and *Nothofagus nervosa* (Phil.) Dimitri & Milano (subgenus *Lophozonia*) were observed at Northern Patagonia (Donoso, Morales & Romero, 1990; Grant & Clement, 2004). The same phenomenon is found within New Zealand species, where the hybridization occurs among them; this has been recorded by Poole (1951) and Poole (1958). In addition, hybridizations have been recorded in cultivation out of natural habitat range for distinct *Nothofagus* species of different continental distribution but always among the same subgenus, that suggests a strong degree of intrinsic reproductive isolation among the different subgenus (Heenan & Smissen, 2013; Acosta & Premoli, 2018). For subgenus *Nothofagus*, the natural hybridization process which has been confirmed for the three evergreen species (Donoso & Atienza, 1983). Quiroga, Vidal & Premoli (2005) confirm the existence of hybridization between *N. antarctica* and *N. pumilio* for forests in Argentina and the possible introgression toward the genetic pool of *N. antarctica*. The natural hybridization between the deciduous species *N. antarctica* and the evergreen *N. dombeyi* was also documented by Stecconi et al. (2004). This proves that there would be incomplete reproductive barriers within the subgenus, even among species with different leaf habit (deciduous and evergreen) (Manos, 1997). The species of the subgenus *Nothofagus* have the potential to hybridize with each other, a fact that has occurred in the past (Premoli et al., 2012). Male and female floral phenologies are synchronized within a given species. However, at a given location, e.g., low elevation, the flowering phenologies of different species may overlap. Therefore, phonological overlap between *N*. *antarctica* with *N. pumilio* at their lower elevational extremes and with *N. dombeyi* at mid elevations may prompt greater opportunities for the potential formation of hybrids (Acosta & Premoli, 2018). On the other hand, the authors have shown that the high conservation of karyotypes found in woody species may account for the presence of the fertile hybrids occurring naturally between species from the same section, clade linage. Karyotype conservation can contribute to explaining the existence of extensive plastid capture that has been observed in wood taxa. The agreement in the distribution of polymorphism of chloroplast DNA of the five species, allowed to determine that they have a shared evolutionary history that dates from the Paleogene, which has been maintained by cycles of hybridization and introgression (Acosta & Premoli, 2010; Premoli et al., 2012; Acosta, Mathiasen & Premoli, 2014). However, the species are morphologically distinguishable from each other, as they are similarly based on molecular polymorphisms through nuclear ITS markers (Acosta & Premoli, 2010). This could be interpreted as the hybridization may have played a fundamental role in favoring the dispersal and recolonization of the species through the flow of interspecific pollen and the permanence of subgenus *Nothofagus* species in environments such as those of Patagonia that have been recurrently affected by disturbances throughout its natural history (Donoso, 1987). Finally, Mathiasen et al. (2017) performed automated sequencing of the ITS nuclear DNA region, for identify different species of subgenus *Nothofagus*. Four of the obtained DNA sequences corresponded to *N. nitida*. They report the finding of *N. nitida* individuals on the eastern face of the Andes in Argentina, escalating its geographic distribution range, delimited from humid areas west of the Andean range, in Chile.

This work, shows a case of hybrid identification in *Nothofagu* s subgenus of different secondary forest of Chile, using high resolution melting with ITS (nuclear Region) and trnL (Chloroplast) approach. Unknown samples of *Nothofagus* subgenus with origins in the coastal and Andean areas of the Los Rios region of Chile were used.

## Material and Methods

### Plant material

**Figure 1:**
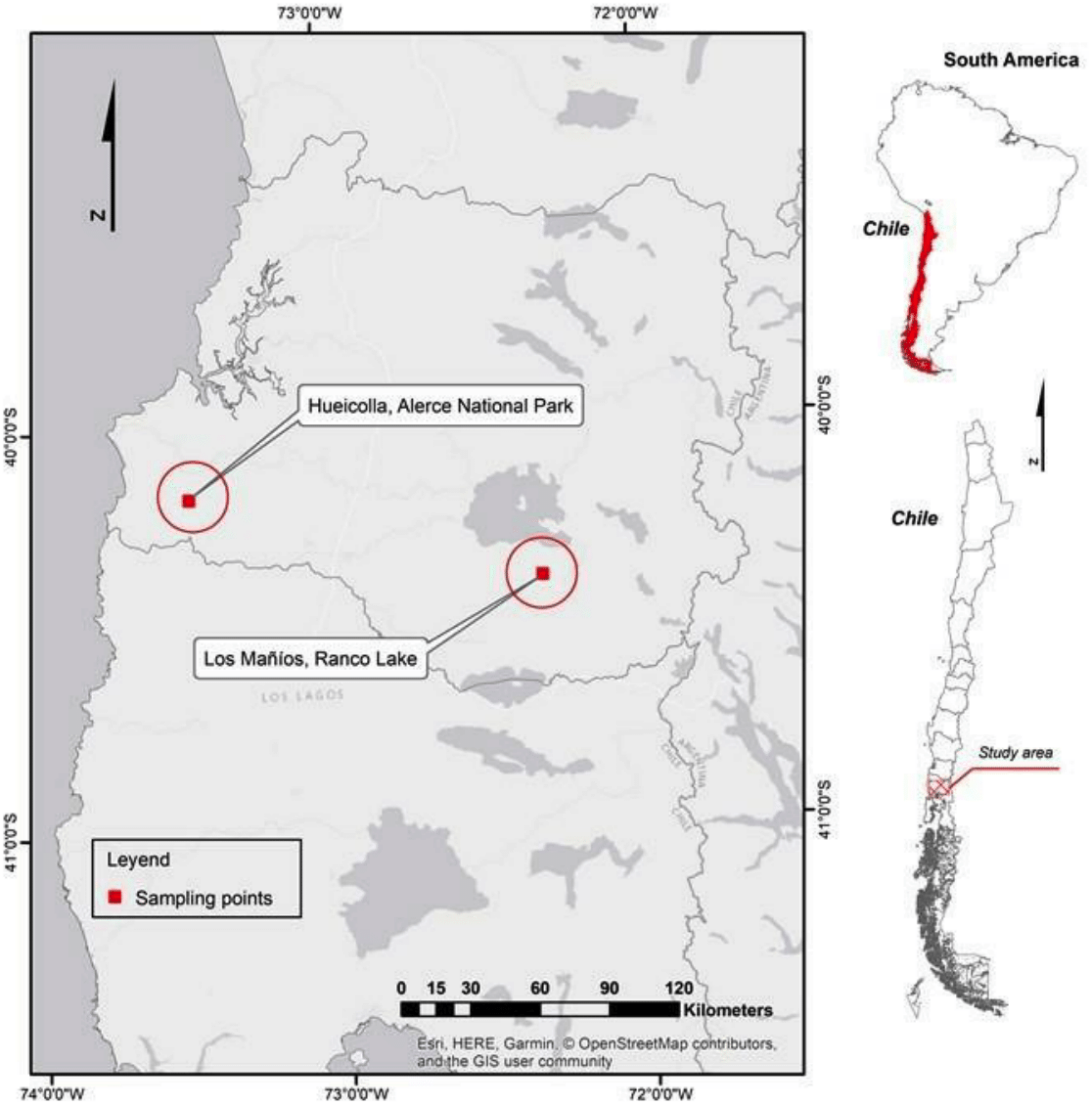
Site of possible hybrids in Los Rios regions in southern Chile. Map of the site of possible hybrids in Los Rios regions in southern Chile.

**Figure 2:**
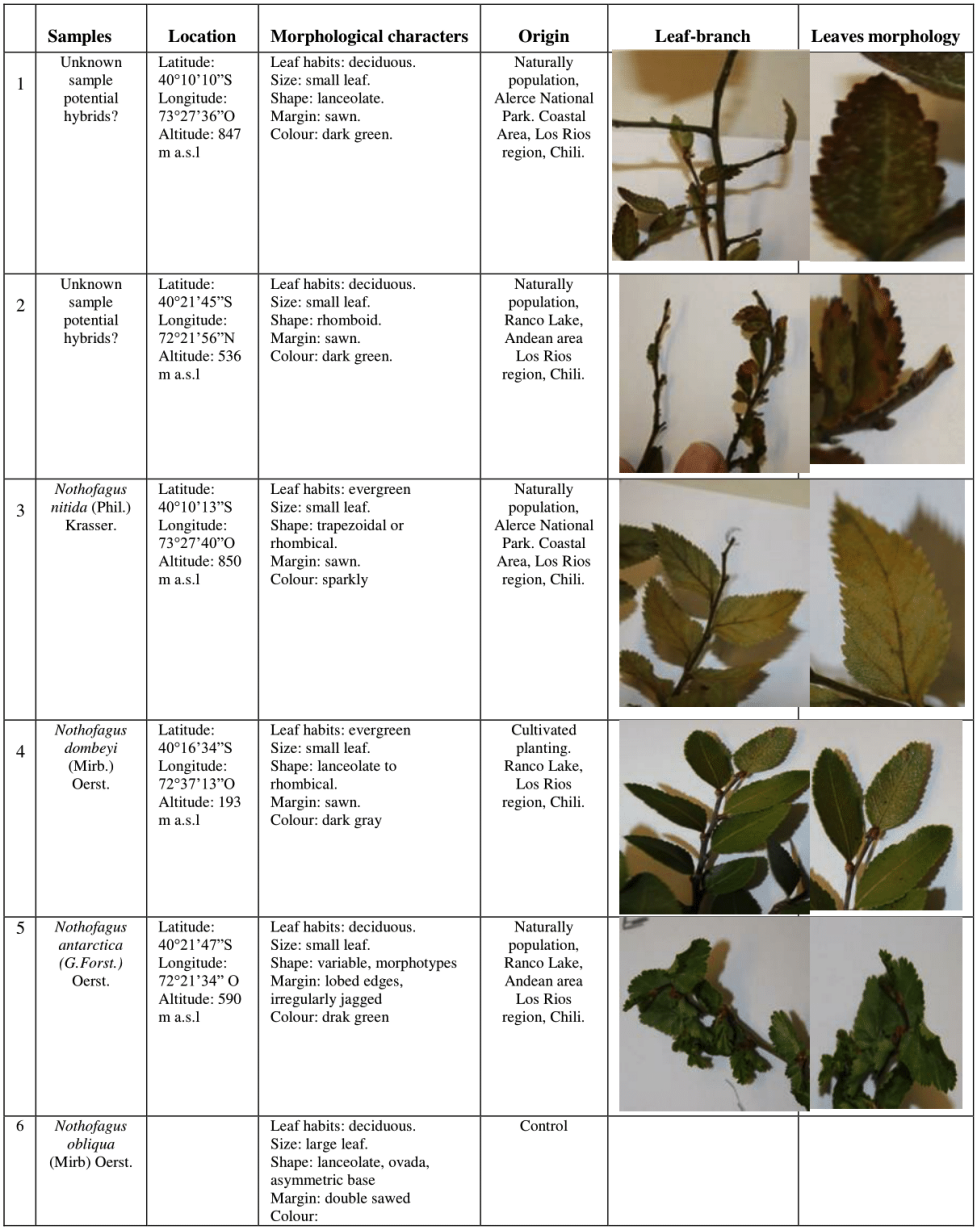
Characteristics and location of studied *Nothofagus* samples. Photographer: Jaime Solano.

Samples corresponding to potential hybrids of secondary forest *Nothofagus*, collected from Los Rios regions in southern Chile (Fig. 1). Plant material was collected from naturally occurring populations. The samples used were: (1) potential hybrid? Coastal area (Alerce National Park) Los Rios region unknown sample; (2) potential hybrid? Andean area (Ranco Lake) Los Rios Region unknown sample. The unknown samples are deciduous; (3) *N. nitida,* in Alerce National Park, Los Rios region; (4) *N. dombeyi,* in Ranco Lake, Los Rios region; (5) *N. antarctica,* in Ranco Lake, Los Rios region; and (6) *N. obliqua* (control sample) (Fig. 2). For unknown individuals, three samples in little stands from secondary forest were selected. The PCR reactions were performed by duplicate. In figures of regions ITS and trnL locus, only one curve of the normalized and derivative of melting is shown in the image in order to improve readability.

### DNA extraction

DNA was extracted from wood and leaves. Genomic DNA was isolated by DNeasy Plant Mini Kit (Qiagen) following the manufacturer’s instructions. It was quantified with a fluorometer Qubit 2.0 (Invitrogen).

### PCR amplification

#### ITS region and trnL locus

PCR amplification was performed in a total volume of 15 µL in Labnet gradient mod. Multigene thermocycler. The reaction mixture contained 20 ng genomic DNA, 1X PCR buffer, 2.0 mM MgCl_2_, 0.2 mM dNTP, 300 nM forward and reverse primers (ITS2 and ITS3; trnL1-F and trnL1-R) (Table 1), and 1 U goTaq flexi DNA polymerase (Promega, USA). For ITS Region, was used initial denaturing step of 94 °C for 5 min followed by 40 cycles of 94 °C for 30 s, 56 °C for 30 s and 72 °C for 45 s, then a final extension step of 72 °C for 10 min. For trnL locus, was used initial denaturing step of 94 °C for 3 min followed by 30 cycles of 95 °C for 20 s, 54 °C for 30 s and 72 °C for 40 s, then a final extension step of 72 °C for 10 min.

#### Electrophoresis of PCR products

Individual PCR products were visualized on agarose gel 2%, in 1x TBE buffer at 100 V for 3 h, stained with ethidium bromide. We used individually isolated duplicate samples for all species.

### HRM-RTPCR

PCR amplification, DNA melting and fluorescence level acquisition for the PCR amplifications were performed in a total volume of 15 µL on an Illumina real-time PCR Thermocycler (ILLUMINA-ECO™ Real-Time PCR System). SYBR Green I was used to monitor the accumulation of the amplified product during PCR and subsequent product melting in an Illumina Thermocycler (Eco™ Software v 4.1.2.0).

### Nuclear DNA (ITS region)

For ITS region, the reaction mix contained 20 ng genomic DNA, 300 nM primers ITS2 and ITS3 (Table 1). Fast PCR Master MIX SYBR Green I 2X (KapaBiosystems, USA). The ITS-PCR protocol was performed using an initial denaturing step of 94 °C for 2 min followed by 40 cycles of 94 °C for 10 s, 56 °C for 30 s and 72 °C for 30 s, then a final extension step of 72 °C for 2 min. The fluorescence data were acquired at the end of each extension step during PCR cycles. Before HRM, the products were denatured at 95 °C for 5 s, and then annealed at 50 °C for 30 s to randomly form DNA duplexes. HRM was performed as follows: pre-melt at the first appropriate temperature for 90 s, and melt at a ramp of 10 °C in an appropriate temperature range with 0.1 °C increments every 2 s. Finally, the 2-resolution melt curve (HRM) of ITS markers was obtained using 95 °C for 15 s, 50 °C for 15 s and 95 °C for 15 s.

**Table 1:** Primers used in the ITS and trnL approach and HRM analysis for *Nothofagus* subgenus.

**Marker**	**Primer**	**Sequence (5′→ 3′)**	**Tm**
ITS region	ITS2 F	5′-ATGCGATACTTGGTGTGAAT-3′	56 °C
	ITS3 R	5′-GACGCTTCTCCAGACTACAAT-3′	56 °C
trnL locus	trnL1-F	5′-CGAAATCGGTAGACGCTACG-3′	54 °C
	trnL1-R	5′-GGGGATAGAGGGACTTGAAC-3′	54 °C

### Chloroplast DNA (trnL locus)

For trnL locus, the reaction mixture contained 20 ng genomic DNA, 300 nM primers trnL1-F and trnL2-R (Table 1). Fast PCR Master MIX SYBR Green I 2X (KapaBiosystems, USA). The trnL-PCR protocol was performed using an initial denaturing step of 94 °C for 3 min followed by 30 cycles of 95 °C for 20 s, 54 °C for 30 s and 72 °C for 40 s, then a final extension step of 72 °C for 2 min. The fluorescence data were acquired same that ITS region. The melting pattern of corresponding sequences of ITS and trnL amplicons from all *Nothofagus* samples were analyzed using the computer program Eco™ Software v4.1.2.0.

### Statistical analysis

Melting Derivate Data for ITS region was used to compare similarity between unknowns samples with *Nothofagus* known species. The first step was to create lower triangular resemblance matrix based on Euclidean distance; later, a hierarchical cluster analysis with single linkage was performed. Once the similar groups were identified, a was carried out distance-based test for homogeneity of multivariate dispersions (PERMDISP), to testing the hypothesis of the existence of homogeneous groups (Anderson, Gorley & Clarke, 2008). Finally, those groups were represented by the Principal Component Analysis (PCA). The statistical analyzes data were done using the following statistical packages: XLSTAT version 4.03 and PRIMER version 7.

## Result

### Nuclear DNA (ITS region)

**Figure 3:**
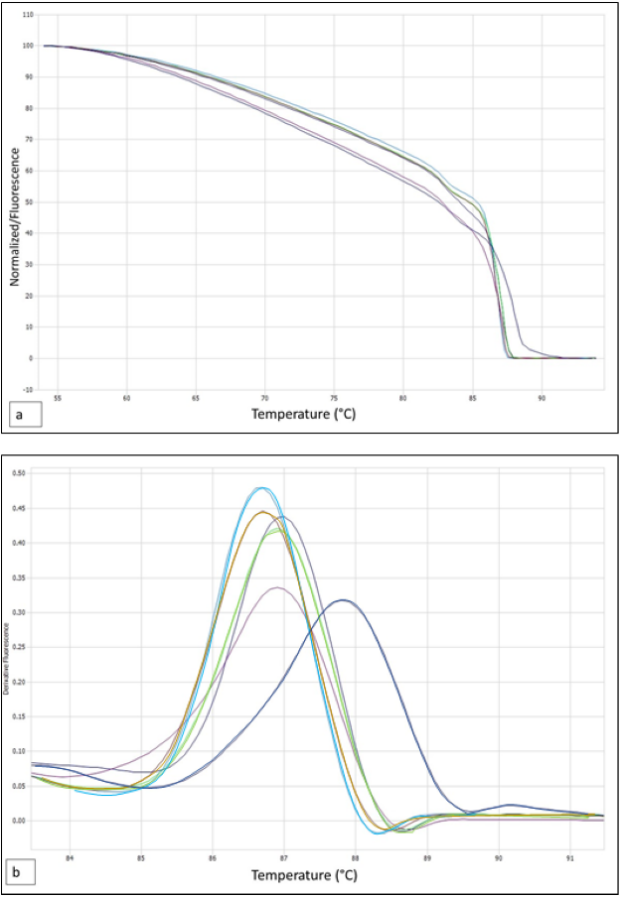
Normalized melt curve and derivative melt with high-resolution (HRM) and ITS approaches. (A) Normalized melt curve (B), Derivative melt with high-resolution (HRM) and ITS approaches. *N. antarctica*, brown; *N. dombeyi*, violet; *N. nitida*, light blue; *N. obliqua*, blue; unknown sample violet and green.

**Figure 4:**
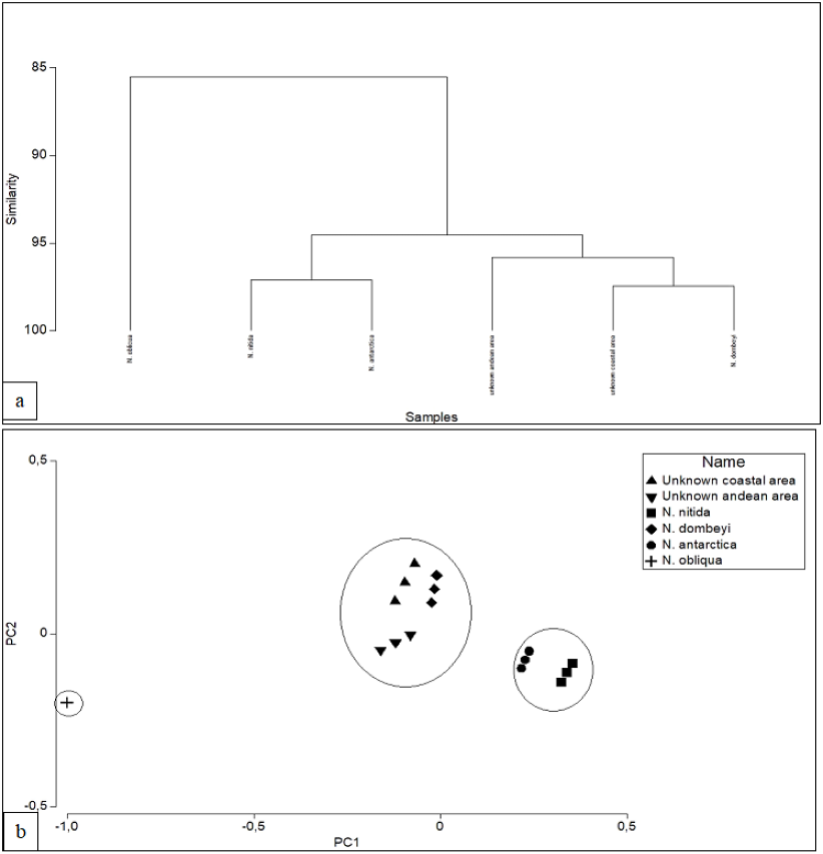
Hierarchical Cluster analysis. (A) Hierarchical cluster analysis with single linkage of the unknowns samples and Nothofagus known species based on Melting Derivate Data for ITS region data. (B) Projection of the unknowns samples and Nothofagus known species on the 1st factor plane from Principal Components analysis. The cumulative explained variance was of 94.3. Line contains homogenius groups with high similarity (PERMDISP).

ITS region shaped polymorphic melting curves for *Nothofagus* samples studied by HRM (Figs. 3A and 3B). Analysis of the normalized HRM curves with ITS region revealed that different species could be distinguished, although the curves show a minimum of difference for each amplicon. Analysis of derivative melt curve, showed curves characteristic for each species based on shape. Peaks were evident for each sample within the range 84.0 °C to 89.0 °C for ITS Region. They were dependent on the interplay between GC content, length of amplified product and sequence. All profiles of *Nothofagus* species produced one peak. ITS region, allowed to distinguish *N. dombeyi* from *N. antarctica*, since they had a ΔTm equal to 0.2 °C (86.9 °C v/s 86.7 °C). In addition, it allowed to distinguish *N. dombeyi* from *N. nitida*, since they had a ΔTm equal to 0.2 °C (86.9° C v/s 86.7 °C). ITS region was not able to distinguish *N. nitida* from *N. antarctica*, since they presented the same Tm (86.7 °C). Unknown samples of *Nothofagus* subgenus, was genetically distinguishable of *N. antarctica*, *N. nitida* and *N. obliqua*, with ITS region. It was not able to distinguish between unknown samples of Andean versus coastal origin since these differences are subtle (Table 2). In addition, the melting curves with ITS approach of the unknown samples, show that both are genetically similar, positioned between *N. dombeyi* and *N. antarctica* and somewhat distant from *N. nitida* (Fig. 4). The unknown samples are genetically very close to *N. dombeyi.* This suggests the presence of hybrid individuals between both species (*N. dombeyi x N. antarctica*) with possible introgression towards the genetic pool of *N. antarctica*, producing the deciduous foliage that both present. The Tm reached by them were 86.95 °C (unknown coastal area) and 86.90 °C (unknown Andean area), with a ΔTm of only 0.05 °C. Based on this, no genetic variation was observed in this genetic region linked to the geographical area of origin, suggesting the hypothesis of individuals hybridization. Both samples are genetically close to *N. dombeyi*, followed by *N. antarctica* and distant from *N. nitida*. All the *Nothofagus* studied: *N. nitida*; *N. dombeyi*; *N. antarctica* and the unknown samples, are clearly distinguishable from *N. obliqua* (sample control), which shows a melting curve with different shape and a Tm value of 87.8 °C, with a ΔTm greater than 1 °C (Table 2). The hierarchical cluster analysis showed three groups that include unknown samples of coastal and Andean origin with *N. dombeyi* (96% similarity), a second that includes *N. antarctica and N. nitida,* differentiating from the previous ones in 14%, and a third group including only *N. obliqua* (Fig. 4A). After verifying the groupings, a test based on the distance for the homogeneity of the multivariate dispersions (PERMDISP) was carried out, showing the homogeneous groups (*p* < 0.05). In Fig. 4B, the first factor plane of the Principal Component Analysis is shown, which is in agreement with the cluster analysis, PERMDISP and HRM analysis. In addition, the foliar morphology of the unknown individuals studied ratified their hybrid condition due to the presence of small leaves with serrated margin and deciduous leaf habit.

### Chloroplast DNA (trnL locus)

**Figure 5:**
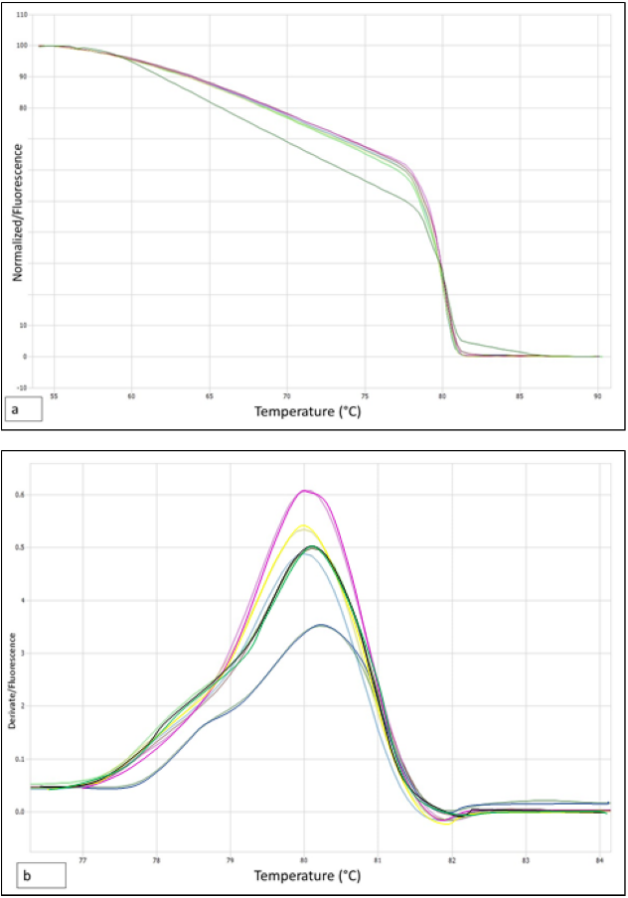
Normalized melt curve and derivative melt with high-resolution melting (HRM) and trnL approaches. (A) Normalized melt curve, (B) derivative melt with high-resolution melting (HRM) and trnL approaches. *N. antarctica*, yellow; *N. dombeyi*, violet; *N. nitida*, black; *N. obliqua*, blue; unknown sample light blue and green.

Analysis of HRM using trnL locus was less polymorphic than ITS region (Figs. 5A and 5B). Analysis of derivative melt curve, presented evident peaks for each sample within the range 77.0 °C to 82.0 °C. This locus was not able to distinguish between *N. dombeyi* and *N. antarctica*, since they had a similar melting curve and equal Tm (80.00 °C). This locus, did not allow to genetically distinguish the unknown samples from the other *Nothofagus* species studied, because the ΔTm were all less than 0.1 °C. This Tm is similar to that shown by *N. obliqua* (sample control); however, it presented a curve of melting in a flattened way that allowed to be distinguished. The melting curve of the samples originating in the coastal area was positioned between *N. dombeyi* and *N. nitida* or between *N. antarctica* and *N. nitida*, a situation that would explain the deciduous behavior (Table 3).

**Table 2:** Fusion temperature variability of amplicons from *Nothofagus* with ITS region approach by HRM analysis.

**Sample–Specie**	**Peak 1(°C) ± SD**
Unknown sample, hybrids?	86.95 ± 0.070
Unknown sample, hybrids?	86.90 ± 0.000
*Nothofagus nitida*	86.70 ± 0.000
*Nothofagus dombeyi*	86.90 ± 0.000
*Nothofagus antarctica*	86.70 ± 0.000
*Nothofagus obliqua*	87.80 ± 0.000

**Table 3:** Fusion temperature variability of the amplicons from *Nothofagus* with trnL region approach by HRM analysis.

**Sample–Specie**	**Peak 1(°C) ± SD**
Unknown sample, hybrids?	80.05 ± 0.070
Unknown sample, hybrids?	80.15 ± 0.070
*Nothofagus nitida*	80.10 ± 0.000
*Nothofagus dombeyi*	80.00 ± 0.000
*Nothofagus antarctica*	80.00 ± 0.000
*Nothofagus obliqua*	80.05 ± 0.212

## Discussion

In recent years, research were focused on DNA barcoding to determine the species of different organisms through sequencing of conserved DNA regions such as internal transcribed spacer (ITS). Another application of DNA barcoding is the use of real-time PCR combined with high resolution melting temperature (HRM) analysis to discriminate specific conserved DNA regions of closely related botanical species. For melting temperature determination, an intercalating fluorescent dye is add to the real time PCR reaction and a derivative melting curve is generated. Distinct nucleotide sequences of a conserved DNA region will provide different melting temperatures (Tm). High-resolution melting (HRM) analysis is a closed-tube method for a rapid analysis of genetic variation within PCR amplicons (Reed & Wittwer, 2004). Genetic variants with changes in the base composition present differences in their melting temperatures. This was detected by monitoring the fluorescence as the temperature is increased, and the species are differentiated by their characteristic melting curves, visualized by the loss of fluorescence as the DNA duplex melts (Ganopoulos et al., 2013; Solano, Anabalon & Encina, 2016). Distinct nucleotide sequences of conserved DNA region will provide different melting temperatures. The HRM technology, characterized nucleic acid samples based on their disassociation behavior and detects small sequence differences in PCR amplified, just by direct melting. Our results, related to the confiscation of hybrids in marijuana used HRM technique, allowed us to discriminated hybrids between *Cannabis sativa* x *Cannabis indica* (unpublished data). However, in the presence of a high degree of kinship or closely related taxa, HRM showed the same limitations when the genetic variation is extremely small (on a single basis) and the selected locus is not sufficiently polymorphic (Sharma et al., 2013). Distinction down to genus and—in many cases—species level is possible based on melting temperatures (Tm) of specific PCR products (Cheng et al., 2006). In our case, the use of high resolution melting with ITS approach allowed the authentication of *Nothofagus* species. Manos (1997), Acosta & Premoli (2010) and Mathiasen et al. (2017), have reported phylogenetic relation with ITS region in Nohofagaceae species. The molecular identification with ITS approach of the individuals of the Andean and coastal area of the Los Ríos region, shows that both are positioned genetically between *N. dombeyi*, and *N. antarctica* and somewhat distant from *N. nitida*. This suggests presence of a hybrid individual between both species (*N. dombeyi* x *N. antarctica*) with possible introgression towards the genetic pool of *N. antarctica*, forging the deciduous foliage of both, situation that has already been showed by Stecconi et al. (2004). This author reports natural hybridization between deciduous (*N. antarctica*) and evergreen (*N. dombeyi*) species. This shows presence of incomplete reproductive barriers within the subgenus even among species with different leaf habits (deciduous and evergreen). For subgenus *Nothofagus*, the natural hybridization process has been confirmed for the three evergreen species (Donoso & Atienza, 1983; Premoli, 1996b; Stecconi et al., 2004; Burns et al., 2010). Quiroga, Vidal & Premoli (2005) confirm the existence of hybridization between *N. antartica* and *N. pumilio* for forests in Argentina, and the possible introgression towards the gene pool of *N. antarctica*. These results are similar to reports of Premoli et al. (2012), who based on ITS nuclear sequences, showed that *N. antarctica* was sister of the monophyletic group containing the three evergreen species (*N. betuloides*, *N. dombeyi* and *N. nitida*). In addition, Acosta & Premoli (2010), report that phylogenetic analysis with ITS region provides strong support for the evergreen species as monophyletic. Among the deciduous form, *N. antarctica* was resolved as a sister to a clade of evergreen species. In the evergreen species, a high rate of relative cross-fertilization has been found (*t* ≥ 0.873) (Premoli, 1996b). Experimental crosses in *N. dombeyi* x *N. nítida* showed that they would be highly self-incompatible (Riveros et al., 1995). The lack of significant differences in the allelic frequencies of the progeny and the presumed parents suggests that the evergreen species conform to a model of mixed reproductive system product of auto and alo-gamia (Premoli, 1996b). Natural hybridization between *N. nervosa* and *N. obliqua* using the Anderson’s Hybrid Index technique, anatomical characteristics of leaves and wood and chromatographic of leaf flavonoid was reported by Donoso, Morales & Romero (1990). There is also some evidence for introgression of *N. nervosa* into *N*. *obliqua* populations. Hybridization between *N. nervosa* (receptor) and *N. obliqua* (pollinator) is another morphological and physiological variation factor for *N. nervosa*. Hybrids F1 are fertile and backcross with both parent species. Analysis of population of *N. obliqua* and *N. nervosa*, show different proportions of F1 hybrids and backcrosses, which would indicate different evolutionary stages in them (Donoso, 2006). Hybridization is a common phenomenon between sympatric species belonging to the same pollen group in *Nothofagus* (Donoso, 1996). Putative hybrids have been recognized between certain combinations of South American species within subgenus *Nothofagus* by Premoli (1996b) and Stecconi et al. (2004). In addition, hybridization is common in families and genera such as *Quercus*, *Nothofagus* and *Pinus* (Donoso, 2004).

The results obtained by trnL locus, did not allow to distinguish between *N. dombeyi* and *N. antarctica*. Previous studies have yielded conflicting topologies for the phylogenetic tree within subgenus *Nothofagus*. A tree based on rbcL cpDNA sequences (Martin & Dowd, 1993) showed that *N. dombeyi* diverged first, but could not resolved the relationships between *N. betuloides*, and *N. pumilio-N. antarctica*, suggesting a close relationship between the deciduous taxa. Dissimilar results between nuclear and chloroplast markers have been reported by Acosta & Premoli (2010). Similar results were reported by Manos (1997) that indicate there is incongruence among chloroplast and nuclear DNA data set. ITS ribotypes illustrate relationships among the delimited species within subgenus *Nothofagus*, the cpDNA phylogeny is geographically structured (Acosta & Premoli, 2010). This means that the species cannot be identified based on cpDNA markers, probably due to the phenomenon of hybridization and “chloroplast capture” because is the mechanism explained below. If crosses of the hybrids with the most abundant parent are continued by introgression, individuals will be produced whose constitution of the nucleus will be that of the pollen-giving species, while conserving the cytoplasm and, consequently, the chloroplasts of the recipient species. Cycles of hybridization-introgression and chloroplast capture among extant and ancestral taxa result in concordant cpDNA phylogeographic patterns, whereas nuclear DNA (ITS) illustrates relationships among delimited species (Acosta & Premoli, 2010). In addition, karyotype conservation can contribute to explaining the existence of extensive “plastid capture” that has been observed in wood taxa (sensu: Acosta & Premoli, 2018).

## Conclusions

This work showed hybrid identification in *Nothofagus* subgenus present within little stands from secondary forests of Chile, using high resolution melting analysis. The ITS region was not able to distinguish between unknown samples of Andean versus coastal origin. In addition, the melting curves with the ITS approach of the unknown samples showed that both were genetically similar, positioned between *Nothofagus dombeyi (Mirb.) Oerst.*, and *Nothofagus antarctica* (G.Forst.) Oerst. and somewhat distant from *Nothofagus nitida* (Phil.) Krasser. This suggests the presence of hybrid individuals between both species (*N. dombeyi* x *N. antarctica*) with possible introgression towards the genetic pool of *N. antarctica*, forging the deciduous foliage that both present. On the other hand, the trnL locus did not allow us to distinguish between the species *N. dombeyi* and *N. antarctica*, since they presented a similar melting curve and equal Tm (80.00 °C). Finally, the trnL locus not allow us to distinguish genetically unknown individuals from others *Nothofagus* species included in this study.

##  Supplemental Information

10.7717/peerj.6779/supp-1 Data S1 Data melting curves Data melting curves for samples analyzed.

## Additional Information and Declarations

### Data Availability

The following information was supplied regarding data availability: The raw data are available in Data S1.
